# Nutritional interventions for the adjunctive treatment of schizophrenia: a brief review

**DOI:** 10.1186/1475-2891-13-91

**Published:** 2014-09-16

**Authors:** Megan Anne Arroll, Lorraine Wilder, James Neil

**Affiliations:** Food for the Brain, 11A Chartfield Avenue, Putney SW15 6DT London; Centre for Nutrition Education & Lifestyle Management, 14A Rectory Road, Wokingham, Berkshire RG40 1DH England

**Keywords:** Schizophrenia, Mental health, Nutrition, Personalised medicine, Adjunct treatment

## Abstract

Schizophrenia is a chronic condition that impacts significantly not only on the individual and family, but the disorder also has wider consequences for society in terms of significant costs to the economy. This highly prevalent condition affects approximately 1% of the worldwide population, yet there are few therapeutic options. The predominant treatment strategy for schizophrenia is anti-psychotic medication (with or without additional talking therapy) even though this approach lacks efficacy in managing the negative symptoms of the condition, is not effective in one-third of the patient group and the side effects of the medication can be severe and debilitating. In recent years, a number of pathophysiological processes have been identified in groups of people with schizophrenia including oxidative stress, one-carbon metabolism and immune-mediated responses. A number of studies have shown that these altered physiological mechanisms can be ameliorated by nutritional interventions in some individuals with schizophrenia. This review briefly describes the aforementioned processes and outlines research that has investigated the utility of nutritional approaches as an adjunct to anti-psychotic medication including antioxidant and vitamin B supplementation, neuroprotective and anti-inflammatory nutrients and exclusion diets. Whilst none of these interventions provides a ‘one-size-fits-all’ therapeutic solution, we suggest that a personalised approach warrants research attention as there is growing agreement that schizophrenia is a spectrum disorder that develops from the interplay between environmental and genetic factors.

**Electronic supplementary material:**

The online version of this article (doi:10.1186/1475-2891-13-91) contains supplementary material, which is available to authorized users.

## Introduction

Schizophrenia is a debilitating condition that affects 1% of the population worldwide [1]. Symptoms of schizophrenia are delineated into positive and negative symptoms; the former include hallucinations, paranoia and delusions, and examples of the latter are reduced motivation, impoverished speech, blunted affect and social withdrawal; in addition this over-arching label can be further sub-grouped based on symptom profile [2]. These symptoms generally emerge in early adulthood and often persist in approximately three-quarters to two-thirds of individuals despite optimal treatment [3–5]. This enduring and/or fluctuating course of illness leads not only to personal distress and disability but also engenders a high societal burden with the estimated total financial cost of £11.8 billion per annum in England alone [6]. Furthermore schizophrenia patients often have comorbid addiction, anxiety and depressive disorders [7], asthma, chronic obstructive pulmonary disease, type-2 diabetes and numerous diabetic-related complications [8] and have increased premature mortality due to underdiagnosed ischemic heart disease and cancer [9, 10]. Indeed, in comparison with the general population those with schizophrenia have a two- to three-fold higher mortality rate, which equates to a 10–25-year reduction in life span [11]. Therefore, there is a need to continually develop and evaluate novel treatments for this disorder, not only for the benefit of the patients but also for the wider society.

Currently, anti-psychotic medication (with or without psychotherapy) is the main treatment for individuals with schizophrenia, an intervention that led to the dopamine (DA) hypothesis. This enduring theory of schizophrenia has evolved over time and was first based upon clinical observations and subsequently empirical evidence from anti-psychotic treatment studies [12]. Briefly, in the 1970s the DA hypothesis initially pinpointed the role of excessive dopamine in schizophrenia [13], which was later refined to include region specificity in terms of prefrontal hypodopaminergia and a subcortical hyperdopaminergia [14]. However, neither of these conceptualisations accounted for the aetiology of dopaminergic abnormality and hence there was a further modification of the DA hypothesis as a ‘final common pathway’ in which numerous genetic and environmental factors can result in increased presynaptic striatal dopaminergic function [15].

Although anti-psychotic medication has persisted as the optimal treatment and is effective in managing the positive symptoms it is limited in terms of treating negative symptoms [16]. In addition to this drawback of drug treatment, this type of therapy is based solely on symptomatology and dosage is often determined by a process of trial and error [17]. In those who respond to anti-psychotic medication, side effects can be distressing and often intolerable; these include involuntary movements such as tremor and rigidity, drug induced Parkinson’s, Tardive dyskinesia, hyper-salivation, increased heart rate, metabolic syndrome and weight gain. Often the side effects themselves require further pharmacological treatment and/or result in treatment discontinuation, leading to subsequent relapse. Furthermore, approximately one-third of individuals with schizophrenia do not respond to anti-psychotic medication, either alone or in conjunction with psychodynamic counselling and other pharmacotherapy [18].

However, there is now increasing evidence that a number of physiological mechanisms such as oxidative stress, one carbon metabolism and atypical immune-mediated responses exist in individuals with schizophrenia, not solely dopaminergic pathophysiology as per the DA hypothesis. Furthermore, these differing pathophysiological manifestations may be ameliorated by nutritional treatment strategies. Therefore, this article aims to briefly outline the aforementioned probable underlying contributory mechanisms that have been observed in patients diagnosed with schizophrenia^a^ and review the research and clinical evidence that has evaluated nutritional interventions in addition to anti-psychotic medication for schizophrenia.

## Method

Only peer-reviewed studies were considered. Human study designs included were Systematic Reviews, Randomised Controlled Trials (double-blinded or otherwise), Cohort studies, Case-Controlled studies and Case Reports. Pubmed, Google Scholar and Science Direct were used to search for relevant studies relating to schizophrenia and nutrition. Additionally, references were followed up and key papers obtained. The initial searches (replicated across all databases) combined schizophrenia with the following terms and their synonyms, alternate spellings and abbreviations:

1. Nutrient Searches: nutrition; Vitamin A, B (all B-Vitamins and synonyms), C, D, E; Omega 3 and Omega 6 Polyunsaturated fatty acids (and all synonyms and abbreviations: e.g. EFA’s, GLA, EPA etc.);
2. Anti-Oxidant Searches: Alpha-lipoic-acid; melatonin
3. Methylation Searches: methylation; Folate; methyfolate; one-carbon metabolism; Vitamin (all B-Vitamins and synonyms); Vitamin C, E;
4. Gluten Searches: Celiac Disease; Gluten Intolerance; Gluten;

By following up the publications of key authors, N-Acetyl Cysteine & Glutathione, and L-Theanine were identified as relevant topics, and following two searches were added:

1. From Ritsner: L-Theanine
2. From Berk, M.S.: N-Acetyl Cysteine; Glutathione

Search terms were combined with logical ANDs as appropriate, and were also combined with terms (with synonyms, abbreviations and alternate spellings) describing study design to narrow search results were appropriate: e.g. Randomised Controlled Trial, Cohort, etc. (Please see Additional file 1 for details of the controlled studies reviewed below and Additional file 2 for nutrient/drug interactions).

### Oxidative stress and the benefits of supplementation

Oxidative stress occurs when the antioxidant defence capacity of an organism is unable to balance the production of reactive oxygen species (ROS) and reactive nitrogen species (RNS), as generated from normal oxidative metabolism [19]. There is an increasing body of evidence demonstrating the occurrence of oxidative stress in those with schizophrenia [20–22]. Overall, current evidence suggests that there is a dysregulation of free radical metabolism, specifically an alteration in the performance of enzymatic and nonenzymatic antioxidant systems in at least a sub-group of people with schizophrenia [20, 22, 23]. Additionally, oxidative stress is associated with a number of pathophysiological mechanisms such as inflammation, mitochondrial dysfunction, lipid peroxidation, DNA damage and apoptosis and hypoactive N-methyl-D-aspartate receptors [22–24]. Due to these findings, the benefit of antioxidant treatment as an adjunct to standard care has been explored in people with schizophrenia, as detailed below.

### N-acetyl cysteine (NAC)

Glutathione (GSH) is an important antioxidant and free radical scavenger that has been found to be decreased in the brains of people with schizophrenia [25, 26]. Although oral GSH supplementation has poor bioavailability [27], N-Acetyl Cysteine (NAC) has been shown to successfully raise plasma glutathione levels in those with schizophrenia [28]. In a case study of a 24-year old woman with chronic and worsening paranoid-type schizophrenia that was generally unresponsive to anti-psychotic treatment, the addition of NAC supplementation improved the patient’s symptomatology in seven days. In addition to the schizophrenia-specific symptoms, improvements were observed in spontaneity, social skills and family relations by both the patient and family members. An RCT including 42 participants with schizophrenia, who were experiencing an acute phase of symptomatology, were randomly assigned to receive up to 2 g/d of NAC plus up to 6 mg/d of risperidone for 8 weeks as an adjunct intervention; significant improvements in negative symptoms were found in the active treatment group comparted to controls but not in positive or general psychopathology [29]. Furthermore, a larger RCT of 140 participants observed significant improvements on global symptomatology, and general and negative symptoms of schizophrenia in the NAC supplementation (2 g/d; in addition to anti-psychotic medication) group in comparison to the placebo group over a 24 week period, but not positive symptoms [30]. Notably, after a 4-week washout period these beneficial effects diminished, with the exception of clinical severity scores.

### Alpha lipoic acid (ALA)

Alpha lipoic acid (ALA) is another powerful antioxidant which crosses the blood brain barrier and performs similar functions to GSH. Early work in the 1950s showed promise with ALA supplementation [31]. More recent research has focused on the side effects of traditional anti-psychotic medication such as weight gain. For instance, Kim et al. [32] proposed that ALA may ameliorate weight gain by modulating adenosine monophosphate-activated protein kinase activity in the hypothalamus and peripheral tissues in those with schizophrenia who are prescribed anti-psychotic therapy as this enzyme is involved with cellular energy homeostasis [33]. In a case series of five individuals with schizophrenia given supplemental ALA, after 12 weeks participants lost a statistically significant amount of weight, total cholesterol levels were reduced and heightened energy was reported by 60% of the sample; however, the symptoms of schizophrenia did not improve from baseline to follow-up [32]. Hence, in this limited data, ALA may be beneficial in counteracting some of the potentially distressing side effects of anti-psychotic medication, which may in turn help with treatment compliance. Furthermore, in a commentary on the topic Seybolt encourages the scientific community to focus on genetic sub-groups (e.g. responders and non-responders) so that targeted ALA treatment may be developed specifically for responders in order to reduce symptomatology and increase functional ability [34].

### Melatonin (N-acetyl-5-methoxytryptamine)

Melatonin (N-acetyl-5-methoxytryptamine) is a naturally occurring compound that plays an important role in the sleep-wake cycle (circadian rhythm) and is also a powerful antioxidant as a direct free radical scavenger and stimulator of antioxidant enzymes [35]. In addition, melatonin enhances intracellular glutathione (GSH) and stabilizes cellular membranes [35]. The nocturnal secretion of melatonin has been found to be decreased in drug-free patients with schizophrenia and this did not improve following anti-psychotic drug treatment [36–38]. This may explain at least partially why insomnia is a commonly reported peripheral symptom in schizophrenia [39]. In terms of supplementation, a small RCT of 19 patients evaluated the effectiveness of melatonin as an addition to standard medical care over seven weeks [40]. It was found that sleep efficiency significantly improved in those receiving the melatonin supplement and furthermore was most effective in people with the worst sleep efficiency. Similarly in a larger RCT of 40 participants, those in the melatonin augmented group reported improved sleep quality and also heightened freshness on awakening, improved mood, and improved daytime functioning [41]. Although these improvements in sleep quality may not directly impact the predominant symptoms of schizophrenia, heightened functioning and improved mood are still notable findings for this difficult-to-treat condition. Finally, in a systematic review of the literature, Anderson and Maes go so far as to suggest that the reduced secretion of melatonin contributes to the aetiology and pathophysiology in patients with schizophrenia and should be considered in treatment approaches not only to manage symptoms but also to control for the metabolic side effects of anti-psychotic medication [42].

The latter is because some anti-psychotics, in particular olanzapine, decrease melatonin which may account for the metabolic dysregulation and weight gain experienced by patients taking these medications [43]. Indeed, in an RCT including both schizophrenia and bipolar disorder patients, melatonin was added to second generation anti-psychotic treatment over an eight-week period to establish whether melatonin could attenuate the adverse metabolic effects produced by these medications [44]. Compared to placebo, the melatonin group saw improvements in diastolic blood pressure and attenuated weight gain, although individuals with bipolar disorder observed the strong beneficial metabolic effects of melatonin on fat mass.

### Vitamins C and E

Vitamins C (ascorbic acid) and E (a-tocopherol) are non-enzymatic dietary antioxidants which may be beneficial in dealing with oxidative stress in schizophrenia as they break free radical-chain reactions [45]. In a double-blind RCT, 40 patients with schizophrenia were tested for serum malondialdehyde (MDA; a marker of lipid peroxidation) and plasma ascorbic acid at baseline and eight weeks following supplementations with vitamin C [46]. High levels of serum MDA and low plasma ascorbic acid were found in the sample as a whole but at follow-up normalisation of these markers were observed only in the group receiving vitamin C as an adjunct to anti-psychotic treatment. Schizophrenic symptomatology also improved significantly in the experimental group compared to those receiving a placebo. Early studies showed promising findings in relation to the benefits of Vitamin E for the treatment of tardive dyskinesia (TD) [47–50]. However a recent meta-analysis of 11 RCTs concluded that there is no evidence that Vitamin E improves established TD; although there were limited findings illustrating that Vitamin E supplementation can prevent TD from deteriorating [51]. Therefore, adding Vitamin E to treatment protocols may still be of some benefit to patients experiencing this distressing symptom that can arise from long-term use of anti-psychotic drugs.

### Essential polyunsaturated fatty acids (PUFAs)

Fatty acids constitute approximately 50-60% of the dry weight of an adult human brain, of which 35% is made up of essential polyunsaturated fatty acids (EPUFAs) [52]. EPUFAs are important components of the phospholipids that comprise specialised cell membranes which play a central role in the physiology and function of the brain [53]. EPUFAs have been hypothesised as an aetiological factor in schizophrenia as early as the 1990s [54] due to findings of low levels of EPUFAs in the red blood cell membranes [55, 56] and brains of those with schizophrenia [57, 58]. In addition to oxidative stress, there have been various other suggestions as to why levels of EPUFAs are decreased in people with schizophrenia including altered neuronal membrane metabolism [59] and/or dysregulation of the inflammatory response system [60]. Even though the exact mechanism behind the reduced levels of EPUFAs is under debate, there have been numerous studies investigating the effectiveness of EPUFA supplementation in patients with schizophrenia. For example, in young adults presenting with sub-threshold psychotic states, a 12-week omega-3 EPUFA supplementation programme consisting of 700 mg of eicosapentaenoic acid (EPA), 480 mg of docosahexaenoic acid (DHA), 220 mg of other omega-3 EPUFAs (18:3n3, 18:4n3, 20:4n3, 21:5n3, and 22:5n3) plus 7.6 mg of mixed tocopherol (Vitamin E) lowered the risk of symptoms developing into a psychotic disorder, as compared to placebo [61]. In older adults with schizophrenia lower levels of red blood cell membrane fatty acids were found at baseline in comparison to controls [62]. These levels increased significantly at 4-month follow-up after omega-3 EPUFA supplementation combined with vitamin C and E and were associated with improvements in psychopathology and quality of life. Importantly in this study, participants with schizophrenia were matched with health controls on age, sex, ethnicity, diet and lifestyle patterns so the differences in baseline levels of EPUFAs were not due to group differences [62]. A recent review has suggested that because levels of fatty acids have been shown to be decreased in the brains of individuals with schizophrenia, and since this type of supplementation exhibits low risk of harm, the addition of EPUFAs to standard medical care for those with schizophrenia may be beneficial [63].

### L-Theanine

L-Theanine (gamma-glutamylethylamide) is an amino acid found in tea plants and may be beneficial due to its antioxidant activity, namely its ability to effectively inhibit peroxidation [64] and diminish the doxorubicin-induced adverse reactions involved in oxidative damage [65]. Furthermore, L-Theanine has also been investigated as an adjunct therapeutic supplement for people with schizophrenia. In an 8-week double-blind randomised placebo-controlled study which included 40 participants diagnosed with schizophrenia or schizoaffective disorder, it was found that compared with placebo, augmentation of antipsychotic therapy with L-Theanine was associated with reduction of anxiety, positive and general psychopathology, compared to placebo [66]. However, negative symptomatology, objective neurocognitive functioning, general functioning, quality of life and side effect prevalence did not differ in the groups. In terms of the mechanism of this supplement, circulating levels of neurochemical indicator brain-derived neurotrophic factor (BDNF) and cortisol-to-dehydroepiandrosterone sulphate (DHEAS)*100 molar ratio appear to be associated with the observed clinical improvements in schizophrenia symptoms, although the exact reason for this relationship is unclear [67]. Miodownik et al. postulate that alterations in serum BDNF level and cortisol-to-DHEAS*100 molar ratio may mediate the beneficial effects of L-Theanine augmentation. As L-Theanine was found to be safe and well tolerated in the trial [66], there appears to be justification for its use; however larger scale studies and replications are warranted to further clarify the role of this amino acid in the treatment of schizophrenia.

### One carbon metabolism and B vitamins

The original observation that certain aberrant methylated compounds can affect mental state was proposed in the 1950s [68] and later refined into the one-carbon cycle hypothesis of schizophrenia [69]. Methylation reactions are now recognised to have an immensely complex influence on biochemical and cellular machinery and represent a widespread biochemical mechanism [70]. Furthermore, elevated levels of homocysteine (a toxic amino acid produced in excess during abnormal methylation processes [71]) have been observed in individuals with schizophrenia [72]. The key areas proposed as factors in the aetiology of schizophrenia with links to one carbon metabolism include faulty/abnormal DNA synthesis, gene regulation, membrane fluidity, synaptic function, and neurotransmitter synthesis [70, 73–75].

### Folate and B vitamin supplementation

The metabolism of folate is a central mechanism in one carbon metabolism where it interacts with the methionine cycle and transmethylation reactions [76]. Studies measuring serum folate levels in patients with schizophrenia have consistently found significantly lower levels in those with the disorder compared to control participants [77–79]. However, these low levels may not be exclusively due to dietary intake as recent work has found associations in variants of four genes linked to folate metabolism where low-functioning genetic variants were associated with increased negative symptom severity [80]. Therefore, Roffman et al. suggest that folate supplementation as an adjunct to anti-psychotic therapy may be of benefit in those with a genetic susceptibility [80]. An early RCT of supplementation with methylfolate in addition to standard treatment illustrated clinical improvements in people with schizophrenia or major depression (33% of which had borderline or definitive folate deficiency) over a 6-month period [81]. In a large scale RCT of 140 participants with schizophrenia, Roffman et al. randomized patients to either folic acid (2 mg) and vitamin B12 (400 mcg) or placebo for 16 weeks. The active treatment group showed improvement in negative symptoms but only when genotypes that were previously associated with negative symptom severity were taken into account. [82]. Similarly, the benefits of folate supplementation on negative symptoms were only revealed once the sample was sub-grouped by genotype; those patients who had at least one copy of the low-functioning variant of the methylenetetrahydrofolate reductase (MTHFR) gene showed a greater improvement in negative symptoms compared to the placebo group. Indeed this variant has been associated with the onset of schizophrenia [83].

Folate supplementation has also been shown to reduce homocysteine levels in individuals with schizophrenia. In a randomised, double-blind, placebo-controlled, crossover design, patients with elevated homocysteine levels were given oral folic acid, B-12, and pyridoxine for three months, followed by a placebo [84]. Homocysteine levels decreased during the supplement phase of the study and were associated with clinical improvements in symptomatology and neurocognitive performance. Therefore, adjunct treatment with B vitamins appears promising in those with high homocysteine levels and also in people with a genetic predisposition to abnormal folate metabolism. Indeed, Roffman et al. [80] propose that genotyping based on specific genes that play crucial roles in the folate metabolism pathway and also broader systems of methylation may lead to targeted nutritional treatment strategies for those with schizophrenia.

### Immune-mediated responses and the therapeutic benefits of casein- and gluten-free diets

Celiac disease (CD) is an immune-mediated condition that leads to inflammation of the small intestinal mucosa resulting in damage, loss of absorptive villi and ultimately nutrient malabsorption [85]. CD is triggered by gliadin, a protein found in wheat gluten, and other alcohol-soluble proteins (prolamines) contained in barley and rye [86]. Elimination of gluten restores intestinal mucosa, resolves symptoms and improves quality of life [86, 87]. The link between schizophrenia and CD in children and young adults was observed in clinical settings as early as the 1950s and 60s [88, 89]. A recent population-based case-controlled study which estimated the lifetime prevalence of a range of autoimmune disorders in schizophrenia found that those with the condition were 3.6 times more likely to be diagnosed with CD than healthy matched controls [90]. In a review of the epidemiological evidence, Kalaydjian et al. noted that the average prevalence of CD in those with schizophrenia across eleven studies was 2.6%, which is higher that the estimated 1% of diagnosed cases of CD in the general population [91]. However, research has identified that the anti-gliadin immune response in schizophrenia may have a different antigenic specificity, being independent of the action of transglutaminase enzyme that is found in those with CD [92]. In some individuals with schizophrenia and other psychiatric conditions such as anxiety, depressive and mood disorders, attention deficit hyperactivity disorder (ADHD) and autism spectrum disorders, CD and gluten sensitivity are possibly involved in the disruption of intestinal permeability and immunologic abnormalities, leading to neurologic and psychiatric symptomatology [93].

### Exclusion diets as an adjunct to anti-psychotic medication

A number of studies have found significant symptom resolution following the introduction of a gluten-free diet for patients with schizophrenia, in addition to standard care. Early work by Dohan found clinical improvements in 62% of male patients who received a milk- and cereal-free diet [94]. The patients on this diet were moved from a locked in-patient setting to an open ward after an average of seven days; in comparison, only 36% of the men on a high-cereal diet were fit to be moved to a less secure setting in this timeframe. A follow-up study replicated these results and also found that those on the milk- and cereal-free diets moved from the locked to the open ward twice as quickly as the patient who consumed high cereal foods [95]. Tellingly, when gluten was added to the experimental diet (without patients’ or staffs’ knowledge) the effects disappeared in both studies. Analogous findings were observed in 14 participants who were given a milk- and cereal-free diet for six weeks and subsequent blind wheat challenge [96]. The improvements in 30 out of 30 measures of psychopathy, social avoidance and participation diminished when gluten was introduced to the diet but returned with the wheat was removed again. However other studies have shown either mixed or non-significant results [97–101], inferring that exclusion diets may only be beneficial for a sub-group of people with schizophrenia. Furthermore, as with all the interventions outlined in this paper, the introduction of exclusion diets should only be considered as an add-on to traditional pharmacological treatment.

### Vitamin D as a risk factor for the development of schizophrenia

It has been hypothesized that low prenatal vitamin D is a risk factor for the incidence of adult-onset schizophrenia as the lack of this fat-soluble vitamin and steroid hormone may detrimentally impact on the developing foetal brain [102]. Research using animal models has shown that low prenatal vitamin D alters brain development [103]. Epidemiological data such as season of birth (higher prevalence of people with schizophrenia have been observed to be born in winter months [104]), urban birth [105], higher rates of schizophrenia in migrant groups [106] and prenatal malnutrition [107] have been cited as support for this hypothesis [108, 109]. In a large-scale birth cohort study of 9,114 people in Finland, the use of vitamin D supplements in the first year of life was associated with a reduced risk of developing schizophrenia at 31 years of age, with higher doses seen as more beneficial than lower amounts [110]. This finding was, however, only observed in men, not women. Conversely in a study looking at women only, those with the highest dietary intake of Vitamin D had a 37% lower risk of developing psychotic-like symptoms compared to women with the lowest intake, when controlling for age, total energy intake, country of birth, BMI and dietary intake of vitamin B12 [111]. However, there is evidence that high doses of vitamin D are not necessarily protective; in 424 matched pairs from a population-based cohort, a bi-modal relationship appeared in which the participants with the lowest and highest levels of vitamin D had increased risk of schizophrenia. Therefore, the relationship between vitamin D and schizophrenia needs further investigation, even though a recent review of the evidence concluded that adequate levels of vitamin D are needed for normal brain development and function and furthermore that at-risk groups should be offered supplementation [112].

## Conclusion

Schizophrenia is a devastating, complex and disabling disorder of which current orthodox treatment (anti-psychotic medication with or without psychodynamic or other forms of talking therapy) has limited efficacy in some patients and is often accompanied by severe side effects which require further medication, leading to poor compliance. This review has highlighted a number of possible contributory mechanisms including oxidative stress, one carbon metabolism, essential fatty acid insufficiency and immune-mediated responses that have been observed in individuals with schizophrenia, and documented the nutritional interventions that have been proposed to modify these aberrations. Nutritional strategies offer promise as an adjunct to pharmacological therapy and pose little risk of harm to patients. However, it must be noted that the likelihood of a ‘one size fits all’ nutritional intervention for the treatment of schizophrenia is slim due to the heterogeneity of the underlying pathophysiology in the condition. Therefore, a more beneficial approach for treating individuals with schizophrenia could be personalised medicine, of which nutritional therapy could play an important part. Within this individualised treatment a patient would be first tested for any deficiencies and physiological abnormalities documented in this paper, with the goal of amending these atypical findings via diet alteration and supplementation. We do not know of any published studies which evaluate such an approach although this type of treatment is offered by specialised private clinics. Future research should attempt to robustly evaluate such a personalised approach as it appears that schizophrenia may be a spectrum of disorders, rather than a discrete disorder with identical biochemical mechanisms and symptomatology, possibly due to the interplay of environmental and genetic influences. Furthermore, additional research aimed at identifying genetic variants associated with disturbances of biological pathways should be carried out to increase our understanding of this complex disorder and guide individualised treatment programmes.

## Endnote

^a^Please note that this review does not claim to review the biochemical and physiological pathways of these mechanisms in depth as this is outside the scope of the article. Please refer to the reference list for further information on each mechanism included in this paper.

## Electronic supplementary material

Additional file 1: Table S1: Characteristics of controlled nutritional treatment studies as an adjunct to antipsychotic medication. (DOCX 29 KB)

Additional file 2: Nutrient/drug interactions.(DOCX 17 KB)

## References

[1] McGrath J, Saha S, Welham J, El Saadi O, MacCauley C, Chant D (2004). A systematic review of the incidence of schizophrenia: the distribution of rates and the influence of sex, urbanicity, migrant status and methodology. BMC Med.

[2] American Psychiatric Association (1994). Diagnostic and Statistical Manual of Mental Disorders 4th edition.

[3] Maurer K, Riecher-R A (1993). The influence of age and sex on the onset and early course of schizophrenia. Br J Psychiatry.

[4] Breier A, Schreiber JL, Dyer J, Pickar D (1991). National Institute of Mental Health longitudinal study of chronic schizophrenia: prognosis and predictors of outcome. Arch Gen Psychiatry.

[5] Petronis A (2004). The origin of schizophrenia: genetic thesis, epigenetic antithesis, and resolving synthesis. Biol Psychiatry.

[6] Schizophrenia Commission (2012). The Abandoned Illness (Schizophrenia Commission Report).

[7] Tsai J, Rosenheck RA (2013). Psychiatric comorbidity among adults with schizophrenia: A latent class analysis. Psychiatry Res.

[8] Schoepf D, Uppal H, Potluri R, Heun R (2014). Physical comorbidity and its relevance on mortality in schizophrenia: a naturalistic 12-year follow-up in general hospital admissions. Eur Arch Psychiatry Clin Neurosci.

[9] Crump C, Winkleby MA, Sundquist K, Sundquist J (2013). Comorbidities and mortality in persons with schizophrenia: a Swedish national cohort study. Am J Psychiatr.

[10] Hennekens CH, Hennekens AR, Hollar D, Casey DE (2005). Schizophrenia and increased risks of cardiovascular disease. Am Heart J.

[11] Laursen TM, Munk-Olsen T, Vestergaard M (2012). Life expectancy and cardiovascular mortality in persons with schizophrenia. Curr Opin Psychiatry.

[12] Toda M, Abi-Dargham A (2007). Dopamine hypothesis of schizophrenia: making sense of it all. Curr Psychiatry Rep.

[13] Snyder SH (1976). The dopamine hypothesis of schizophrenia: focus on the dopamine receptor. Am J Psychiatry.

[14] Davis KL, Kahn RS, Ko G, Davidson M (1991). Dopamine in schizophrenia: a review and reconceptualization. Am J Psychiatry.

[15] Howes OD, Kapur S (2009). The dopamine hypothesis of schizophrenia: version III—the final common pathway. Schizophr Bull.

[16] Buchanan RW, Freedman R, Javitt DC, Abi-Dargham A, Lieberman JA (2007). Recent advances in the development of novel pharmacological agents for the treatment of cognitive impairments in schizophrenia. Schizophr Bull.

[17] Bentall RP (2009). Doctoring the Mind: Why Psychiatric Treatments Fail.

[18] Ritsner MS (2010). Is a Neuroprotective Therapy Suitable for Schizophrenia Patients? Brain Protection in Schizophrenia, Mood and Cognitive Disorders.

[19] Kohen R, Nyska A (2002). Oxidation of biological systems: oxidative stress phenomena, antioxidants, redox reactions, and methods for their quantification. Toxicol Pathol.

[20] Fendri C, Mechri A, Khiari G, Othman A, Kerkeni A, Gaha L (2005). Oxidative stress involvement in schizophrenia pathophysiology: a review. L’Encéphale.

[21] Zhang M, Zhao Z, He L, Wan C (2010). A meta-analysis of oxidative stress markers in schizophrenia. Sci China Life Sci.

[22] Bitanihirwe BK, Woo T-UW (2011). Oxidative stress in schizophrenia: an integrated approach. Neurosci Biobehav Rev.

[23] Ciobica A, Padurariu M, Dobrin I, Stefanescu C, Dobrin R (2011). Oxidative stress in schizophrenia-focusing on the main markers. Psychiatr Danub.

[24] Wood SJ, Yücel M, Pantelis C, Berk M (2009). Neurobiology of schizophrenia spectrum disorders: the role of oxidative stress. Ann Acad Med Singapore.

[25] Yao JK, Leonard S, Reddy R (2006). Altered glutathione redox state in schizophrenia. Dis Markers.

[26] Gawryluk JW, Wang J-F, Andreazza AC, Shao L, Young LT (2011). Decreased levels of glutathione, the major brain antioxidant, in post-mortem prefrontal cortex from patients with psychiatric disorders. Int J Neuropsychopharmacol.

[27] Witschi A, Reddy S, Stofer B, Lauterburg B (1992). The systemic availability of oral glutathione. Eur J Clin Pharmacol.

[28] Lavoie S, Murray MM, Deppen P, Knyazeva MG, Berk M, Boulat O, Bovet P, Bush AI, Conus P, Copolov D, Fornari E, Meuli R, Solida A, Vianin P, Cuénod M, Buclin T, Do KQ (2008). Glutathione precursor, N-acetyl-cysteine, improves mismatch negativity in schizophrenia patients. Neuropsychopharmacology.

[29] Farokhnia M, Azarkolah A, Adinehfar F, Khodaie-Ardakani M-R, Hosseini S-M-R, Yekehtaz H, Tabrizi M, Rezaei F, Salehi B, Sadeghi S-M-H, Moghadam M, Gharibi F, Mirshafiee O:, Akhondzadeh S (2013). N-acetylcysteine as an adjunct to risperidone for treatment of negative symptoms in patients with chronic schizophrenia: a randomized, double-blind, placebo-controlled study. Clin Neuropharmacol.

[30] Berk M, Copolov D, Dean O, Lu K, Jeavons S, Schapkaitz I, Anderson-Hunt M, Judd F, Katz F, Katz P, Ording-Jespersen S, Little J, Conus P, Cuenod M, Do KQ, Busha AI (2008). N-acetyl cysteine as a glutathione precursor for schizophrenia—a double-blind, randomized, placebo-controlled trial. Biol Psychiatry.

[31] Giamattei L (1957). Thioctic acid in therapy of schizophrenia. Osp Psichiatr.

[32] Kim E, Park D-W, Choi S-H, Kim J-J, Cho H-S (2008). A preliminary investigation of α-lipoic acid treatment of antipsychotic drug-induced weight gain in patients with schizophrenia. J Clin Psychopharmacol.

[33] Foufelle F, Ferré P (2005). Role of adenosine monophosphate-activated protein kinase in the control of energy homeostasis. Curr Opin Clin Nutr Metab Care.

[34] Seybolt SE (2010). Is it time to reassess alpha lipoic acid and niacinamide therapy in schizophrenia?. Med Hypotheses.

[35] Reiter RJ, Acuna-Castroviejo D, Tan DX, Burkhardt S (2001). Free radical-mediated molecular damage. Mechanisms for the protective actions of melatonin in the central nervous system. Ann N Y Acad Sci.

[36] Monteleone P, Maj M, Fusco M, Kemali D, Reiter RJ (1992). Depressed nocturnal plasma melatonin levels in drug-free paranoid schizophrenics. Schizophr Res.

[37] Monteleone P, Natale M, La Rocca A, Maj M (1997). Decreased nocturnal secretion of melatonin in drug-free schizophrenics: no change after subchronic treatment with antipsychotics. Neuropsychobiology.

[38] Fanget F, Claustrat B, Dalery J, Brun J, Terra J-L, Marie-Cardine M, Guyotat J (1989). Nocturnal plasma melatonin levels in schizophrenic patients. Biol Psychiatry.

[39] Monti JM, Monti D (2005). Sleep disturbance in schizophrenia. Int Rev Psychiatry.

[40] Shamir E, Laudon M, Barak Y, Anis Y, Rotenberg V, Elizur A, Zisapel N (2000). Melatonin improves sleep quality of patients with chronic schizophrenia. J Clin Psychiatry.

[41] Suresh KP, Andrade C, Bhakta SG, Singh NM (2007). Melatonin in schizophrenic outpatients with insomnia: a double-blind, placebo-controlled study. J Clin Psychiatry.

[42] Anderson G, Maes M (2012). Melatonin: an overlooked factor in schizophrenia and in the inhibition of anti-psychotic side effects. Metab Brain Dis.

[43] Dodd S, Maes M, Anderson G, Dean OM, Moylan S, Berk M (2013). Putative neuroprotective agents in neuropsychiatric disorders. Prog Neuro-Psychopharmacol Biol Psychiatry.

[44] Romo‒Nava F, Alvarez‒Icaza González D, Fresán‒Orellana A, Saracco Alvarez R, Becerra‒Palars C, Moreno J, Ontiveros Uribe MP, Berlanga C, Heinze G, Buijs RM (2014). Melatonin attenuates antipsychotic metabolic effects: an eight‒week randomized, double‒blind, parallel‒group, placebo‒controlled clinical trial. Bipolar Disord.

[45] Mahadik SP, Scheffer RE (1996). Oxidative injury and potential use of antioxidants in schizophrenia. Prostaglandins Leukot Essent Fat Acids.

[46] Dakhale G, Khanzode S, Khanzode S, Saoji A (2005). Supplementation of vitamin C with atypical antipsychotics reduces oxidative stress and improves the outcome of schizophrenia. Psychopharmacology.

[47] Adler LA, Peselow E, Rotrosen J, Duncan E, Lee M, Rosenthal M, Angrist B (1993). Vitamin E treatment of tardive dyskinesia. Am J Psychiatry.

[48] Lohr JB, Caligiuri MP (1996). A double-blind placebo-controlled study of vitamin E treatment of tardive dyskinesia. J Clin Psychiatry.

[49] Lohr JB, Cadet JL, Lohr MA, Larson L, Wasli E, Wade L, Hylton R, Vidoni C, Jeste DV, Wyatt RJ (1988). Vitamin E in the treatment of tardive dyskinesia: the possible involvement of free radical mechanisms. Schizophr Bull.

[50] Elkashef AM, Wyatt RJ (1999). Tardive dyskinesia: possible involvement of free radicals and treatment with vitamin E. Schizophr Bull.

[51] Soares-Weiser K, Maayan N, McGrath J (2011). Vitamin E for neuroleptic-induced tardive dyskinesia.

[52] Wainwright PE (2002). Dietary essential fatty acids and brain function: a developmental perspective on mechanisms. Proc Nutr Soc.

[53] McNamara RK, Carlson SE (2006). Role of omega-3 fatty acids in brain development and function: potential implications for the pathogenesis and prevention of psychopathology. Prostaglandins Leukot Essent Fat Acids.

[54] Horrobin DF, Glen AIM, Vaddadi K (1994). The membrane hypothesis of schizophrenia. Schizophr Res.

[55] Peet M, Laugharne J, Horrobin D, Reynolds G (1994). Arachidonic acid: a common link in the biology of schizophrenia?. Arch Gen Psychiatry.

[56] Peet M, Laugharne J, Rangarajan N, Horrobin D, Reynolds G (1995). Depleted red cell membrane essential fatty acids in drug-treated schizophrenic patients. J Psychiatr Res.

[57] Horrobin DF, Manku MS, Hillman H, Iain A, Glen M (1991). Fatty acid levels in the brains of schizophrenics and normal controls. Biol Psychiatry.

[58] Yao JK, Leonard S, Reddy RD (2000). Membrane phospholipid abnormalities in postmortem brains from schizophrenic patients. Schizophr Res.

[59] Assies J, Lieverse R, Vreken P, Wanders RJ, Dingemans PM, Linszen DH (2001). Significantly reduced docosahexaenoic and docosapentaenoic acid concentrations in erythrocyte membranes from schizophrenic patients compared with a carefully matched control group. Biol Psychiatry.

[60] Das UN (2013). Polyunsaturated fatty acids and their metabolites in the pathobiology of schizophrenia. Prog Neuro-Psychopharmacol Biol Psychiatry.

[61] Amminger GP, Schafer MR, Papageorgiou K, Klier CM, Cotton SM, Harrigan SM, Mackinnon A, McGorry PD, Berger GE (2010). Long-chain {omega}-3 fatty acids for indicated prevention of psychotic disorders: a randomized, placebo-controlled trial. Arch Gen Psychiatry.

[62] Arvindakshan M, Ghate M, Ranjekar PK, Evans DR, Mahadik SP (2003). Supplementation with a combination of ω-3 fatty acids and antioxidants (vitamins E and C) improves the outcome of schizophrenia. Schizophr Res.

[63] Akter K, Gallo DA, Martin SA, Myronyuk N, Roberts RT, Stercula K, Raffa RB (2012). A review of the possible role of the essential fatty acids and fish oils in the aetiology, prevention or pharmacotherapy of schizophrenia. J Clin Pharm Ther.

[64] Yokozawa T, Dong E (1997). Influence of green tea and its three major components upon low-density lipoprotein oxidation. Exp Toxicol Pathol.

[65] Sugiyama T, Sadzuka Y (2004). Theanine, a specific glutamate derivative in green tea, reduces the adverse reactions of doxorubicin by changing the glutathione level. Cancer Lett.

[66] Ritsner MS, Miodownik C, Ratner Y, Shleifer T, Mar M, Pintov L, Lerner V (2011). L-theanine relieves positive, activation, and anxiety symptoms in patients with schizophrenia and schizoaffective disorder: an 8-week, randomized, double-blind, placebo-controlled, 2-center study. J Clin Psychiatry.

[67] Miodownik C, Maayan R, Ratner Y, Lerner V, Pintov L, Mar M, Weizman A, Ritsner MS (2011). Serum levels of brain-derived neurotrophic factor and cortisol to sulfate of dehydroepiandrosterone molar ratio associated with clinical response to l-theanine as augmentation of antipsychotic therapy in schizophrenia and schizoaffective disorder patients. Clin Neuropharmacol.

[68] Osmond H, Smythies J (1952). Schizophrenia: a new approach. Br J Psychiatry.

[69] Smythies JR, Alarcon RD, Bancroft AJ, Monti JA, Morere DA, Tolbert LC, Walter-Ryan WG (1986). Role of the one-Carbon Cycle in Neuropsychiatry. Biological Methylation and Drug Design.

[70] Krebs M, Bellon A, Mainguy G, Jay T, Frieling H (2009). One-carbon metabolism and schizophrenia: current challenges and future directions. Trends Mol Med.

[71] Scott JM, Weir DG (1998). Folic acid, homocysteine and one-carbon metabolism: a review of the essential biochemistry. J Cardiovasc Risk.

[72] Muntjewerff J-W, Kahn RS, Blom HJ, den Heijer M (2006). Homocysteine, methylenetetrahydrofolate reductase and risk of schizophrenia: a meta-analysis. Mol Psychiatry.

[73] Frankenburg FR (2007). The role of one-carbon metabolism in schizophrenia and depression. Harv Rev Psychiatry.

[74] Regland B (2005). Schizophrenia and single-carbon metabolism. Prog Neuro-Psychopharmacol Biol Psychiatry.

[75] Sugden C (2006). One-carbon metabolism in psychiatric illness. Nutr Res Rev.

[76] Mattson MP, Shea TB (2004). Folate and homocysteine metabolism in neural plasticity and neurodegenerative disorders. Trends Neurosci.

[77] Goff DC, Bottiglieri T, Arning E, Shih V, Freudenreich O, Evins AE, Henderson DC, Baer L, Coyle J (2004). Folate, homocysteine, and negative symptoms in schizophrenia. Am J Psychiatr.

[78] Muntjewerff J-W, van der Put N, Eskes T, Ellenbroek B, Steegers E, Blom H, Zitman F (2003). Homocysteine metabolism and B-vitamins in schizophrenic patients: low plasma folate as a possible independent risk factor for schizophrenia. Psychiatry Res.

[79] Lerner V, Kanevsky M, Dwolatzky T, Rouach T, Kamin R, Miodownik C (2006). Vitamin B12 and folate serum levels in newly admitted psychiatric patients. Clin Nutr.

[80] Roffman JL, Brohawn DG, Nitenson AZ, Macklin EA, Smoller JW, Goff DC (2013). Genetic variation throughout the folate metabolic pathway influences negative symptom severity in schizophrenia. Schizophr Bull.

[81] Godfrey PSA, Toone BK, Bottiglien T, Laundy M, Reynolds EH, Carney MWP, Flynn TG, Chanarin I (1990). Enhancement of recovery from psychiatric illness by methylfolate. Lancet.

[82] Roffman JL, Lamberti J, Achtyes E, Macklin EA, Galendez GC, Raeke LH, Silverstein NJ, Smoller JW, Hill M, Goff DC (2013). Randomized multicenter investigation of folate plus vitamin b12 supplementation in schizophrenia. JAMA Psychiatry.

[83] Gilbody S, Lewis S, Lightfoot T (2007). Methylenetetrahydrofolate reductase (MTHFR) genetic polymorphisms and psychiatric disorders: a HuGE review. Am J Epidemiol.

[84] Levine J, Stahl Z, Sela B-A, Ruderman V, Shumaico O, Babushkin I, Osher Y, Bersudsky Y, Belmaker RH (2006). Homocysteine-reducing strategies improve symptoms in chronic schizophrenic patients with hyperhomocysteinemia. Biol Psychiatry.

[85] Dieterich W, Ehnis T, Bauer M, Donner P, Volta U, Riecken EO, Schuppan D (1997). Identification of tissue transglutaminase as the autoantigen of celiac disease. Nat Med.

[86] Fasano A, Catassi C (2001). Current approaches to diagnosis and treatment of celiac disease: an evolving spectrum. Gastroenterology.

[87] Mustalahti K, Lohiniemi S, Collin P, Vuolteenaho N, Laippala P, Maki M (2002). Gluten-free diet and quality of life in patients with screen-detected celiac disease. Eff Clin Pract.

[88] Bender L (1953). Childhood schizophrenia. Psychiatry Q.

[89] Graff H, Handford A (1961). Celiac syndrome in the case histories of five schizophrenics. Psychiatry Q.

[90] Eaton WW, Byrne M, Ewald H, Mors O, Chen C-Y, Agerbo E, Mortensen PB (2006). Association of schizophrenia and autoimmune diseases: linkage of Danish national registers. Am J Psychiatr.

[91] Kalaydjian A, Eaton W, Cascella N, Fasano A (2006). The gluten connection: the association between schizophrenia and celiac disease. Acta Psychiatr Scand.

[92] Samaroo D, Dickerson F, Kasarda DD, Green PHR, Briani C, Yolken RH, Alaedini A (2010). Novel immune response to gluten in individuals with schizophrenia. Schizophr Res.

[93] Jackson JR, Eaton WW, Cascella NG, Fasano A, Kelly DL (2012). Neurologic and psychiatric manifestations of celiac disease and gluten sensitivity. Psychiatry Q.

[94] Dohan FC, Grasberger J, Lowell F, Johnston H, Arbegast AW (1969). Relapsed schizophrenics: more rapid improvement on a milk-and cereal-free diet. Br J Psychiatry.

[95] Dohan F, Grasberger J (1973). Relapsed schizophrenics: earlier discharge from the hospital after cereal-free, milk-free diet. Am J Psychiatry.

[96] Singh MM (1976). Wheat gluten as a pathogenic factor in schizophrenia. Science.

[97] Vlissides DN, Venulet A, Jenner F (1986). A double-blind gluten-free/gluten-load controlled trial in a secure ward population. Br J Psychiatry.

[98] Potkin SG, Weinberger D, Kleinman J, Nasrallah H, Luchins D, Bigelow L, Linnoila M, Fischer SH, Bjornsson TD, Carman J, Gillin JC, Wyatt RJ (1981). Wheat gluten challenge in schizophrenic patients. Am J Psychiatry.

[99] Storms LH, Clopton JM, Wright C (1982). Effects of gluten on schizophrenics. Arch Gen Psychiatry.

[100] Osborne M, Crayton JW, Javaid J, Davis JM (1982). Lack of effect of a gluten-free diet on neuroleptic blood levels in schizophrenic patients. Biol Psychiatry.

[101] Rice JR, Ham CH, Gore WE (1978). Another look at gluten in schizophrenia. Am J Psychiatry.

[102] McGrath J (1999). Hypothesis: is low prenatal vitamin D a risk-modifying factor for schizophrenia?. Schizophr Res.

[103] Eyles D, Brown J, Mackay-Sim A, McGrath J, Feron F (2003). Vitamin d3 and brain development. Neuroscience.

[104] Bradbury TN, Miller GA (1985). Season of birth in schizophrenia: a review of evidence, methodology, and etiology. Psychol Bull.

[105] Torrey EF, Bowler AE, Clark K (1997). Urban birth and residence as risk factors for psychoses: an analysis of 1880 data. Schizophr Res.

[106] Cantor-Graae E, Selten J-P (2005). Schizophrenia and migration: a meta-analysis and review. Am J Psychiatr.

[107] Xu M-Q, Sun W-S, Liu B-X, Feng G-Y, Yu L, Yang L, He G, Sham P, Susser E, St Clair D, He L (2009). Prenatal malnutrition and adult schizophrenia: further evidence from the 1959–1961 Chinese famine. Schizophr Bull.

[108] Eyles DW, Burne TH, McGrath JJ (2013). Vitamin D, effects on brain development, adult brain function and the links between low levels of vitamin D and neuropsychiatric disease. Front Neuroendocrinol.

[109] McGrath JJ, Burne TH, Féron F, Mackay-Sim A, Eyles DW (2010). Developmental vitamin D deficiency and risk of schizophrenia: a 10-year update. Schizophr Bull.

[110] McGrath J, Saari K, Hakko H, Jokelainen J, Jones P, Järvelin M-R, Chant D, Isohanni M (2004). Vitamin D supplementation during the first year of life and risk of schizophrenia: a Finnish birth cohort study. Schizophr Res.

[111] Hedelin M, Löf M, Olsson M, Lewander T, Nilsson B, Hultman CM, Weiderpass E (2010). Dietary intake of fish, omega-3, omega-6 polyunsaturated fatty acids and vitamin D and the prevalence of psychotic-like symptoms in a cohort of 33 000 women from the general population. BMC Psychiatry.

[112] McCann JC, Ames BN (2008). Is there convincing biological or behavioral evidence linking vitamin D deficiency to brain dysfunction?. FASEB J.

