# Supplementary material for: Nutritional interventions for the adjunctive treatment of schizophrenia: a brief review
Source: Nutr J. 2014 Sep 16;13:91. doi: 10.1186/1475-2891-13-91 (PMC4171568; doi:10.1186/1475-2891-13-91)
Supplement: Supplementary file 1 — Additional file 1: Table S1: Characteristics of controlled nutritional treatment studies as an adjunct to antipsychotic medication. (DOCX 29 KB) [file 12937_2014_827_MOESM1_ESM.docx]

| **Study** | **Diagnosis** | **N** | **Total daily dosage** | **Study design** | **Outcome measures** | **Outcome** |
| --- | --- | --- | --- | --- | --- | --- |
| N-Acetyl Cysteine (NAC) |  |  |  |  |  |  |
| Farokhnia et al 2013 | Schizophrenia | N=42 | 2 g/d NAC & 6 mg/d of risperidone | 8 week randomized, double-blind, placebo-controlled, parallel-group study | PANSS (Positive and Negative Symptoms Scale) | NAC therapy resulted in a safe and effective strategy for reducing negative symptoms |
| Lavoie et al 2008 | Schizophrenia | N=11 | 2 g/day for 60 days and then placebo for another 60 days (or vice versa) | 8 week double blind crossover design | Mismatched negativity and plasma glutathione concentration | Significant improvements in mismatch negativity in NAC group.  Plasma glutathione levels were increased following NAC treatment |
| Berk et al 2008 | Schizophrenia | N=140 | 2 g/d | 6 month double blind placebo controlled trial | PANSS, CGI, GAF, SOFAS, BAS, Simpson-Angus Scale and the abnormal involuntary Movements Scale | Improvements seen in negative symptoms based on PANSS: improvements also seen on CGI and BAS.  Improvements were lost at 1 month follow up visit |
| Alpha lipoic acid (ALA) |  |  |  |  |  |  |
| Kim et al 2008 | Schizophrenia | N=5 | 1200 mg/day orally | 12 week | Brief Psychiatric Rating Scale and the Montgomery-Asberg Depression Rating Scale | Although after 12 weeks cholesterol levels reduced and energy was reported to have increased by 60%, symptoms of schizophrenia did not improve. |
| Melatonin (N-acetyl-5-methoxytryptamine) |  |  |  |  |  |  |
| Shamir et al 2000 | Schizophrenia | N=19 | 2 mg | 7 week randomized, double-blind, cross-over, clinically based trial ([melatonin](http://europepmc.org/abstract/MED/10847313/?whatizit_url_Chemicals=http://www.ebi.ac.uk/chebi/searchId.do?chebiId=CHEBI%3A16796) or placebo for 2 treatment periods of 3 weeks each with 1 week washout between treatment periods) | Urine collection, Actigraphy - Activity- and rest-derived [sleep](http://europepmc.org/abstract/MED/10847313/?whatizit_url_go_term=http://www.ebi.ac.uk/ego/GTerm?id=GO:0030431) parameters | Sleep efficiency significantly improved in those receiving the melatonin supplement and furthermore was most effective in people with the worst sleep efficiency. |
| Suresh at al 2007 | Schizophrenia individuals with insomnia | N=40 | 3-12 mg per night | 15 day double-blind study | Questionnaires which assessed of aspects of [sleep](http://europepmc.org/abstract/MED/17335321/?whatizit_url_go_term=http://www.ebi.ac.uk/ego/GTerm?id=GO:0030431) functioning;  [sleep](http://europepmc.org/abstract/MED/17335321/?whatizit_url_go_term=http://www.ebi.ac.uk/ego/GTerm?id=GO:0030431)-onset latency, heightened freshness on awakening, improved daytime functioning and improved mood | Improved sleep quality and also heightened freshness on awakening, improved mood, and improved daytime functioning. Although these improvements in sleep quality may not directly impact the predominant symptoms of schizophrenia, heightened functioning and improved mood |
| Roma-Nava et al 2014 | Schizophrenia with second generation anti-psychotic treatment | N=44  N=24 | 5mg per day | 8 week randomized, double‐blind, parallel‐group, placebo‐controlled clinical trial | weight, blood pressure, lipid, glucose, body composition, and anthropometric measures | Improvements in diastolic blood pressure and attenuated weight gain, although individuals with bipolar disorder observed the strong beneficial metabolic effects of melatonin on fat mass. |
| Vitamins C and E |  |  |  |  |  |  |
| Dakhale et al 2005 | Schizophrenia (on atypical antipsychotics medication) | N=40 | 500 mg/day | 8 week prospective, double-blind, placebo-controlled, noncrossover | Increased serum Malondialdehyde (MDA), plasma ascorbic acid levels and brief psychiatric rating scale (BPRS) score. | High levels of serum MDA and low plasma ascorbic acid were found in the sample as a whole but at follow-up normalisation of these markers were observed only in the group receiving vitamin C group as an adjunct to anti-psychotic treatment. Schizophrenic symptomatology also improved significantly in the experimental group compared to those receiving a placebo. |
| Adler et al 1993 | Schizophrenia with tardive dyskinesia | N=28 (27 of which have schizophrenia) | Vitamin E (maximal dose 1,600 IU/day) | 8-22 week double-blind, parallel-group comparison study | The Abnormal Involuntary Movement Scale scores | Significant improvement was found in the Abnormal Involuntary Movement Scale scores for those treated with Vitamin E |
| Lohr et al 1996 | Schizophrenia with tardive dyskinesia | N=35 | Vitamin E  (800 IU b.i.d) | 8 week double-blind placebo-controlled study | Several measures to assess abnormal involuntary movement, parkinsonism and psychopathology and measures of tardive dyskinesia | Treatment of vitamin E resulted in reduced levels of severity of tardive dyskinesia. Involuntary movement scale score was reduced and positive symptoms for those with schizophrenia were reduced. |
| Lohr et al 1988 | Schizophrenia with tardive dyskinesia | N=15 | Vitamin E | Randomized crossover design | Abnormal Involuntary Movements Scale (AIMS) / Brief Psychiatric Rating Scale (BRRS)/ Simpson-Angus Scale (SAS) for Extrapyramidal Side Effects | Reduction in AIMS score. Decrease in BPRS. No change in SAS |
| Essential polyunsaturated fatty acids (PUFAs) |  |  |  |  |  |  |
| Amminger et al 2010 | Prodromal adolescents | N=81 | 840 mg EPA and 700 mg DHA  /d | 12 week Randomized, double-blind, placebo-controlled trial – followed by 40 week monitoring | Transition to psychiatric disorder / symptomatic and functional changes | Lowered the risk of symptoms developing into a psychotic disorder, as compared to placebo |
| Arvindakshan M et al 2003 | Schizophrenia (on medication) | N=28 | Mixture EPA/DHA (180:120 mg) & vitamins E/C (400iu:500mg) | 4 months – pre treatment, post treatment and 4 month post treatment | Control (=45), PANSS | Significant improvements on most of the clinical measures, sustained for additional 4 months of supplement free period |
| L-Theanine |  |  |  |  |  |  |
| Ritsner et al 2011 | Schizophrenia or schizoaffective disorder | N=40 (20 did not complete the study) | 400mg/d of L-theanine added to ongoing antipsychotic treatment | 8-week, randomized, double-blind, placebo-controlled, 2-center study |  | Reduction of anxiety, positive and general psychopathology. However, negative symptomatology, objective neuro-cognitive functioning, general functioning, quality of life and side effect prevalence did not differ in the groups. |
| Miodownik et al 2011 | Schizophrenia or schizoaffective disorder | N=40 | 400 mg/d | 8-week, double-blind, randomized, placebo-controlled trial | Brain-derived neurotrophic factor (BDNF), dehydroepiandrosterone (DHEA), its sulfate (DHEAS), cortisol, cholesterol, and insulin | Observed clinical improvements in schizophrenia symptoms, although the exact reason for this relationship is unclear |
| Folate and B vitamin supplementation |  |  |  |  |  |  |
| Godfrey et al 1990 | Acute schizophrenics with borderline folate deficiency | N=41 | 15mg methylfolate daily for 6 months + psychotropic meds. | 6 month double blind, placebo controlled trial |  | Patients sig. improved clinical and social recovery, greater with time |
| Roffman, Lamberti, Achtyes et al 2013 | Schizophrenia | N=440 | folic acid (2 mg) and vitamin B12 (400 mcg) | 16 week randomised, placebo controlled trial |  | Improvement in negative symptoms but only when genotypes that were previously associated with negative symptom severity were taken into account. |
| Levine et al 2006 | Chronic schizophrenia patients inpatients with >15μmol/l | N=42 | 2mg folic acid + 25mg pyridoxine + 400μB12 per day or placebo as add-on | 6 month randomised, double-blind, placebo-controlled, crossover design | PANSS & AIMS baseline, 3 months and 6 months. Plasma Hcy taken monthly | Sig. difference in reduced PANSS at 3 months. |
| Exclusion diets as an adjunct to anti-psychotic medication |  |  |  |  |  |  |
| Vlissides, Venulet & Jenner, 1986 | Psychotic disorders, particularly schizophrenia | N=24 | Gluten-free v gluten-containing diet | 14 week double-blind, placebo control trial | Psychotic In-Patient profile (PIP) | Beneficial changes found between pre-trial and gluten-free time in 5 aspects of PIP. |
| Potkin et al 1981 | Young individuals with chronic schizophrenia | N=8 | 30 g of gluten for 5 weeks and a placebo challenge for 8 weeks | 13 week double-blind, placebo control trial | BPRS / Serum alpha 1 acid glycoprotein measurement | No reduction in BPRS. No inflammatory response (Serum). Sensitivity to dietary gluten not found. |
| Storms, Clopton & Wright, 1982 | Schizophrenia | N=26 | gluten-free peanut-flour supplementary cookies | 10 day double-blind, placebo control trial | Profile on Mood States | No improvement found in experimental group. In fact the gluten-added group showed greater improvement in tension-anxiety and anger-hostility |
