# Supplementary material for: Nutritional interventions for the adjunctive treatment of schizophrenia: a brief review
Source: Nutr J. 2014 Sep 16;13:91. doi: 10.1186/1475-2891-13-91 (PMC4171568; doi:10.1186/1475-2891-13-91)
Supplement: Supplementary file 2 — Additional file 2: Nutrient/drug interactions.(DOCX 17 KB) [file 12937_2014_827_MOESM2_ESM.docx]

***Nutrient/drug interactions***

**Before prescribing/taking any dietary supplement it is important to check that it does not interact with any other medication/supplementation that the patient is currently taking.  Be aware there maybe potential interactions that need to be explored.**

**The information below was derived from the Natural standard database (**[**www.naturalstandard.com**](http://www.naturalstandard.com/)**), the Office of Dietary Supplements, National Institutes of Health (**[**http://ods.od.nih.gov**](http://ods.od.nih.gov/)**) and Natural Medicines Comprehensive Database (**[**http://natural**](http://natural/) [**database.therapeuticresearch.com**](http://database.therapeuticresearch.com/)**).**

**Please be aware this is based on generic supplements, and each brand of supplement and specific quantity should be checked individually.**

***Antioxidants*:**

***N-acetyl cysteine*: some NAC contains Vitamin C, vitamin C has been hypothesized to antagonize the effects of reactive oxygen species-generating *antineoplastic* drugs.**

**MODERATE interaction with ACTIVATED CHARCOAL**

**MAJOR interaction with NITROGLYCERINE**

***Melatonin* – currently not available in the UK**

**MODERATE interaction with ANTICOAGULANT/ANTIPLATELET drugs**

**MODERATE interaction with ANTIDIABETES Drugs**

**MODERATE interaction with BENZODIAZEPINES**

**MODERATE interaction with CAFFEINE**

**MAJOR interaction with CNS DEPRESSANTS**

**MODERATE interaction with CONTRACEPTIVE Drugs**

**MINOR interaction with FLUMAZENIL**

**MODERATE interaction with FLUVOXAMINE**

**MODERATE interaction with IMMUNOSUPPRESSANTS**

**MODERATE interaction with NIFEDIPINE GITS**

**MODERATE interaction with VERAPAMIL**

***Essential polyunsaturated fatty acids*:**

**EPUFAs: n-3 fatty acids is a synonym for omega-3 fatty acids, fish oil, and alpha-linolenic acid. In theory, omega-3 fatty acids may increase the risk of bleeding when taken with herbs and supplements that are believed to increase the risk of bleeding.  Numerous other agents may theoretically increase the risk of bleeding, although this has not been proven in most cases.**

**MODERATE interaction with ANTICOAGULANT/ANTIPLATELET drugs**

**MODERATE interaction with ANTIHYPERTENSIVE drugs.**

***B vitamins***

***B2 – riboflavin*:**

**MINOR interaction with ANTICHOLINERGIC Drugs**

**MINOR interaction with PHENOBARBITAL**

**MINOR interaction with PROBENECID**

**MINOR interaction with TRICYCLIC ANTIDEPRESSANTS**

***B3 – niacin*:**

**MODERATE interaction with ALCOHOL**

**MODERATE interaction with ALLOPURINOL**

**MODERATE interaction with ANTIDIABETES drugs**

**MINOR interaction with ASPIRIN**

**MODERATE interaction with BILE ACID SEQUESTRANTS**

**MODERATE interaction with CARBAMAZEPINE**

**MODERATE interaction with CLONIDINE**

**MODERATE interaction with HMG-CoA REDUCTASE INHIBITORS (“Statins”)**

**MODERATE interaction with PRIMIDONE**

**MODERATE interaction with PROBENECID**

**MODERATE interaction with SULFINPYRAZONE**

**MINOR interaction with TRANSDERMAL NICOTINE**

***B6 – pyridoxine* : vitamin B6 can interact with certain medications, and several types of medications might adversely affect vitamin B6 levels.  *Antiepileptic drugs*: some antiepileptic drugs including valproic acid (sometimes given to patients with schizophrenia) increase the catabolism rate of vitamin B6 vitamers, resulting in low plasma PLP concentrations and hyperhomocysteinemia.  High homocysteine levels in antiepileptic drug users might increase the risk of epileptic seizures and systemic vascular events, including stroke, and reduce the ability to control seizures in patients with epilepsy.**

**MODERATE interaction with *AMIODARONE***

**MINOR interaction with *LEVODOPA***

**MODERATE interaction with *PHENOHARBITAL***

**MODERATE interaction with *PHENYTOIN***

***B9 – Folate*.  Folate/folic acid supplements can interact with several medications including medications used to treat *cancer and antiepileptic* drugs and advice should be given from appropriate healthcare professionals.**

**MODERATE interaction with 5-FLUOROURACIL**

**MODERATE interaction with CAPECITABINE**

**MODERATE interaction with FOSPHENYTOIN**

**MODERATE interaction with METHOTREXATE**

**MODERATE interaction with PHENOBARBITAL**

**MODERATE interaction with PHENYTOIN**

**MODERATE interaction with PRIMIDONE**

**MODERATE interaction with PYRIMETHAMINE**

***B12 – Cobalamin*. Vitamin B12 is involved in the recycling of folate coenzymes through involvement in methionine synthesis.  B12 is also needed for nerve myelination, and prolonged deficiency leads to irreversible neurological damage.  Food sources include almost all animal products and certain algae and bacteria.  The most common reason for deficiency is a failure of intrinsic factor secretion.   It has extremely low toxicity, with no toxic effects seen in man.  Certain drugs interact with vitamin B12 by reducing levels in the body including certain *antibiotics, proton pump inhibitors and Metformin*, used to treat diabetes.**

**SEVERE interactions with *CHLORAMPHENICOL* – do not take this combination.**

***Vitamin D* – There are no known drug interactions.**
